# Dual-Task-Based Drum Playing with Rhythmic Cueing on Motor and Attention Control in Patients with Parkinson’s Disease: A Preliminary Randomized Study

**DOI:** 10.3390/ijerph181910095

**Published:** 2021-09-26

**Authors:** Jin-Kyoung Park, Soo Ji Kim

**Affiliations:** 1 Music Therapy Education, Graduate School of Education, Ewha Womans University, Seoul 03760, Korea; ppjk7314@naver.com; 2 Laboratory of Brain & Cognitive Sciences for Convergence Medicine, Hallym University College of Medicine, Anyang 14068, Korea

**Keywords:** Parkinson’s disease, dual-task, rhythmic cueing, rehabilitation, drum playing

## Abstract

Although there have been increasing reports regarding the effectiveness of dual-task interventions in rehabilitation, the scope of this research is limited to gross motor movement, such as gait among patients with Parkinson’s disease (PD). To expand the dual-task paradigm to upper extremity motor and attention control in PD, drum playing with modulation of musical elements was attempted. The objective of this study was to evaluate the effects of a drum playing intervention with rhythmic cueing on upper extremity motor control and attention control in patients with PD. Twelve participants were randomly assigned to the drum playing intervention with rhythmic cueing group or the control group. The results showed that the drum playing with rhythmic cueing (DPRC) group significantly increased their sustained time of entrainment (45 BPM) and their latency time until entrainment from pretest to posttest. For the DPRC group, the latency time until entrainment was significantly improved, and improvements in cognitive measures were also found. This study shows that DPRC has great potential to improve upper extremity motor control and attention control and supports the development of new interventions that include this technique for rehabilitation in patients with PD.

## 1. Introduction

Parkinson’s disease (PD) is a progressive neurodegenerative disorder that results in motor symptoms involving difficulty with movement initiation and disruptions in movement control [1] as well as nonmotor symptoms [2] involving impairments in memory, attention, and executive function [3]. Most of the motor symptoms of PD, such as tremor, rigidity, bradykinesia, and movement freezing [4], are caused by a dopaminergic deficiency in the basal ganglia, including the loss of dopamine cells in the basal nucleus [5]. Even a 50% loss in dopamine cells has been associated with involuntary motor disorders due to decreased functioning of the central nervous system [6]. In turn, decreased motor control affects the trajectory, timing, and consistency of movement and leads to difficulties with stretch and grab movements in daily living [7,8,9].

A deficiency of dopamine in the basal ganglia is also associated with neural network disruption resulting in cognitive impairment [5]. Almost 40% of PD patients experience cognitive decline [10], and decline in cognitive performance is associated with decreased social interaction, decreased participation in activities of daily living (ADL) [11,12,13,14,15], and greater risk of mortality [16]. As expected, limited motor and cognitive functioning makes it more difficult to engage in ADL which contributes to decreased quality of life among patients with PD [13].

In previous studies, performing a motor-cognitive dual task requires increased cognitive loads resulting in increases of gait variabilities [17], reduces automaticity, and reduces cognitive resources [18,19]. Attention control is critical in dual-task performance to produce coordinated functional movements [20,21,22]. Brain imaging studies show that dual-task performance requires connectivity with several brain regions, including both motor and cognitive areas [19,23], which leads to greater efficiency in performing dual tasks. Due to the neural connectivity associated with dual tasks, a wide range of dual-task training protocols (i.e., walking while talking, walking while performing calculations) has been developed that show positive effects on complex motor functioning with cognitive loads [24].

Recently, a rhythm-motor task has been proposed as a new paradigm to enhance dual-task performance because rhythmic cueing can play a role in mediating cognitive loads in a motor-cognitive dual task, [25], which is especially important for older adults with cognitive aging, decrements in gait integrity [26], and increased tendency for falls [27]. Research in this area has focused on the mechanism of auditory-motor interaction in dual-task performance involving upper arm movement [25,26,27,28,29,30,31]. In particular, playing rhythmic patterns using two hands requires activation of motor and cognitive networks to induce successful task performance [32]. Because the strength and timing of arm and hand movements vary during drum playing [33], drum playing of rhythmic patterns can improve bimanual coordination and accuracy of movement [34].

Rhythmic cueing has long been employed in PD rehabilitation to compensate for reduced movement automaticity due to dopaminergic denervation in the basal ganglia [19]. Movement automaticity in dual-task execution is particularly aggravates in relation to frontal executive dysfunction among patients with PD [35]. It shows that entrainment of brain oscillations to rhythmically cued movement results in behavioral advantages for patients with PD [36]. By coupling regular rhythmic cues to motor tasks, dual task could be successfully performed by allocating attention at anticipated time points [37] and directing attention to the concurrent task [38]. Recently, an fMRI study found greater internetwork connectivity between the auditory network and the executive control network of the brain modulating activity in cortico-cerebellar networks [39]. This was interpreted as the compensatory role of auditory cues in enhancing dual-task performance of PD patients by reducing dual-task interference [39]. These results highlight the potential effectiveness of using rhythmic cueing in dual-task training for improved motor and cognitive functioning among PD patients.

In addition to rhythm perception through rhythmic cueing, rhythm production in a form of drum playing can be primary task in the dual-task paradigm because it requires the recruitment of attentional resources for movement execution. Successful motor control of the upper limbs is based on integration of cognitive, sensory, and motor information with timely response [40,41]. Considering PD-induced impaired automaticity, which is observed even in well-practiced movements [42,43], rhythmic cueing with drum playing can serve as repetitive upper arm movement by using an attentional control strategy.

Given the implications of previous studies, we hypothesized that PD patients participating in dual-task-based drum playing with rhythmic cueing (DPRC) would demonstrate greater improvements in motor control, Nine Hole Peg Test (NHPT) scores, and attention control compared to a control group. This preliminary study examined the effects of DPRC on the motor and cognitive functioning of patients with PD. In addition to the rehabilitative effects, it was expected that patients with PD would enjoy the social aspects of the group drum playing experience.

## 2. Materials and Methods

### 2.1. Participants

Twenty patients with PD, ages 40–60 years, were recruited from local community centers and online PD support groups. Eligibility criteria included a Stage II or III diagnosis on the Hoehn and Yahr (H&Y) scale [44] and a score over 22 on the Korean version of the mini-mental state examination (K-MMSE) [45]. Additionally, participants had to be able to independently move without the use of a gait orthosis and be free of any hearing loss diagnosis. Of the 20 initially identified participants, four did not meet the inclusion criteria, and four others were excluded from analyses because they dropped out of the intervention for health reasons.

### 2.2. Procedure

Study participants were provided with a detailed description of the procedures to be followed in the study and written informed consent prior to their participation. The informed consent form included a description of the program content, the duration of the study, the procedures to be used, the expected benefits and risks of participation in the study, and confidentiality. Additionally, we provided participants with information regarding the study aims and methods and their right to withdraw anytime without reprisal. The researcher who evaluated the results was not involved in the intervention and had no knowledge of which group the participants had been assigned to in order to prevent any possible judgment or manipulation of the results during the evaluations. The study was conducted according to the Declaration of Helsinki principles and reports the required information accordingly.

**Figure 1:**
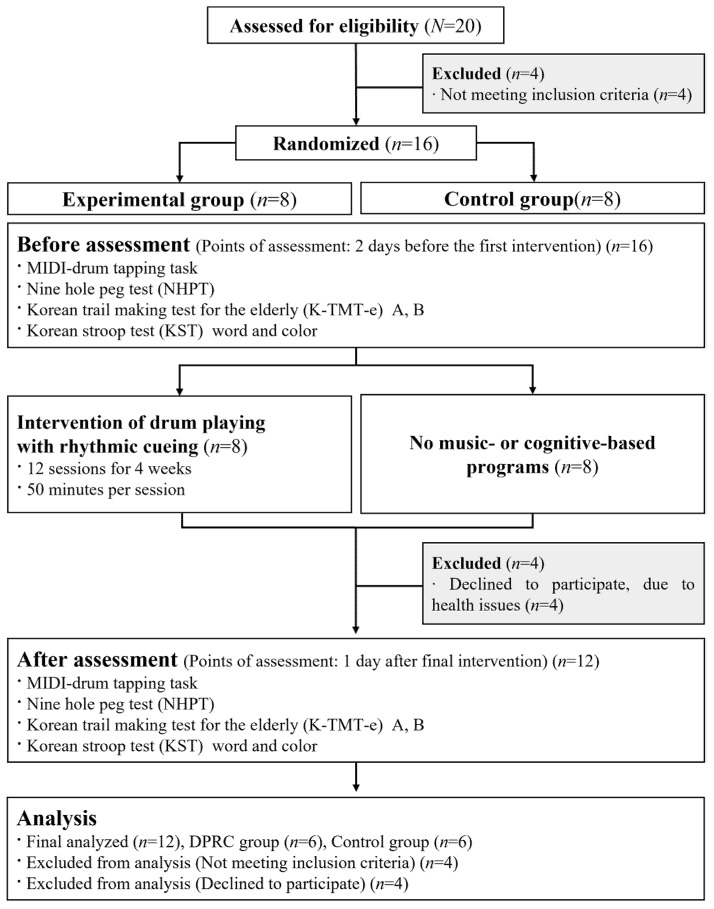
Flowchart for group allocation.

Participants who volunteered to participate were randomly assigned to either the experimental group or the control group. The random assignment procedure was performed with numbers generated by a program of Microsoft Office Excel (Microsoft, Redmond, WA, USA). Of the 20 initially identified participants, four did not meet the inclusion criteria, and four others were excluded from analyses because they dropped out of the study due to health reasons. As a result, 16 people were included in the two groups, then, after 4 dropouts, 12 people, six in each group, were analyzed (see Figure 1). Three 50-min group sessions were held each week for 12 weeks, and assessments were conducted before and after the intervention. During the intervention, the control group attended regular programs, such as gait rehabilitation and speech therapy at the community center, and participants of the control group did not attend any music- or cognitive-based programs.

### 2.3. Measurements

**Figure 2:**
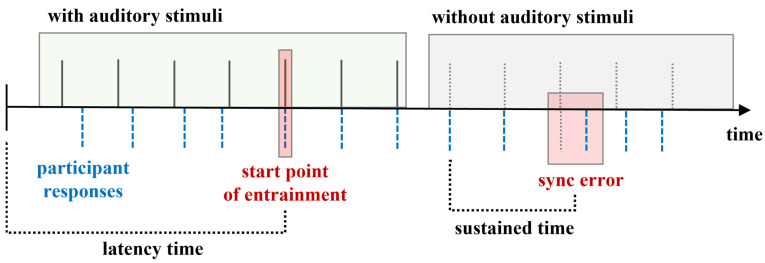
The measurement paradigm of the MIDI drum tapping task. The latency time until the entrainment refers to time from cueing until each participant’s tapping interval started synchronizing. The sustained time of entrainment refers to time until each participant’s tapping became regular without rhythmic cueing.

In this study, the musical instrument digital interface (MIDI) drum(ALESIS, Cumberland, RI, USA) tapping task [46] was used to evaluate motor control function during bimanual tapping. The tapping consisted of two task conditions: drum tapping with and without cueing (see Table 1). In the first stage, participants were asked to tap a drum to a self-paced tempo for 15 s. with both hands simultaneously as regularly as possible. The time from when the direction to start was provided until each participant started tapping was the latency time until self-paced tapping became regular. Then, they were provided with rhythmic cueing at regular intervals for 45 and 105 BPM for 15 s and were instructed to tap their drum in as equal intervals as possible to the regular rhythmic cueing. The time from the presence of cueing until each participant tapped their drum was the latency time until entrainment for 45 and 105 BPM. Next, participants were provided with changes in tempo. In this condition, the BPM of regular rhythmic cueing was changed from 45 to 105 BPM and from 105 to 45 BPM. Additionally, participants were instructed to shift their drum tapping as soon as possible after a tempo change. The time from 45 to 105 BPM or 105 to 45 BPM until entrainment was the latency time for changes in tempo. Finally, the participants were instructed to continue tapping without rhythmic cueing and with rhythmic cueing removed at 15 s after the starting point to measure sustained time for movement entrainment. Sustained time of entrainment was measured for 45 and 105 BPM (see Figure 2). In addition, the NHPT [47] was used to measure fine motor function, as this score is affected by muscle strength, tactile sensitivity of the thumb, and presence of intention tremor. The NHPT [47] requires participants to repeatedly place and then remove nine pegs into nine holes, one at a time, as quickly as possible. The Korean trail making test for the elderly (K-TMT-e) measures visual attention, processing speed, mental flexibility, and executive function [48]. Part A of the K-TMT-e [48] asks participants to connect 15 numbered circles in sequential order, and Part B asks them to connect 15 circles alternating between sequential numbers and words (e.g., 1-Monday-2-Thusday). In this study, completion times on the K-TMT-e [48] were used to assess attention control. The Korean Stroop Test (KST) [49] assesses the ability to inhibit cognitive interference. This interference occurs when the processing of a stimulus feature affects the simultaneous processing of another attribute of the same stimulus [50]. In the KST [49], participants are required to read two different tables. First, the WR condition requires participants to read the names of colors printed in black ink. Second, the CR condition requires participants to name the color of words printed in different colored ink (for instance, the word “red” is printed in green ink).

### 2.4. Intervention of Drum Playing with Rhythmic Cueing

The intervention consisted of the bimanual exercise of drum playing to strengthen motor control. The musical structure of the DPRC intervention gave a temporal cue for movement timing during the participants’ musical performance, and rhythmic cueing was provided as a stimulus to activate cognitive processing during musical performance. The DPRC intervention consisted of five stages (see Table 2).

The first stage involved playing with regular rhythmic cueing in which drum playing occurred following a metronome beat of music with regular tempo and repeated musical structure. The second stage involved playing the drum according to a changing tempo. At this stage, the rhythmic cueing alternated between 45 and 105 BPM with predictable structure. Common to both stages was the demand for exact motor function and sustained attention while playing the DPRC. Participants had to sustain their auditory attention to track the rhythmic cueing, and this sustained attention caused entrainment between auditory functioning and upper extremity motor functioning. Next, the third stage involved sustaining drum playing according to self-selected rhythmic cueing: simultaneous presentation of regular rhythmic cueing by metronome or unpredictable melodic cueing by electronic piano. Unpredictable melodic cueing consisted of dynamic syncopation and irregular rhythm patterns. In particular, during Stage 3, participants needed to exhibit inhibitory control of selective attention. During drum playing to regular rhythmic cueing, participants needed to sustain constant movement patterns and inhibit dynamic unpredictable melodic cueing. In Stage 4, participants switched between stop and initiation according to two different regular rhythmic cueing patterns played by touch bells: one pattern included stable beat intervals while the other one had a syncopated rhythm pattern. Stage 5 involved switching between drum playing and singing according to the same rhythmic cueing patterns used in Stage 4. Participants initiated drum playing when a stable rhythmic pattern was introduced by touch bells, and participants switched tasks from drum playing to singing when the rhythmic pattern changed. The processes required during Stages 4 and 5 were the most demanding of the intervention, equally requiring high levels of attention and motor control. In other words, the task switching activity following rhythmic cueing was a dual task, requiring divided attention to simultaneously perform the cognitive processing and exercise tasks triggered by external auditory stimuli.

### 2.5. Data Collection and Analysis

Drum tapping data were collected through the following steps. First, the latency time until regularly self-paced tapping measured the time interval from the verbal starting cue until participants began tapping their drum. Second, the latency time until entrainment for 45 and 105 BPM in response to auditory stimulation was measured via synchronization errors in the drum-tapping task. The synchronization errors were calculated as the time interval between stimulation and response during self-paced tapping. For the mean synchronization errors, the timing of the tap following the onset of rhythmic cueing was calculated and averaged for each trial. Third, the sustained time of entrainment for 45 and 105 BPM was calculated for the tapping time without rhythmic cueing (see Figure 2). Finally, the NHPT score measured the execution time of each hand. Parts A and B of the K-TMT-e were scored based on task completion time. The KST was scored based on the completion time of each task. For each task, average and variability (standard deviation) were calculated to measure significance of completion times and task scores. Data were analyzed using the Statistical Package for the Social Sciences (SPSS 22.0) software, and the data were analyzed descriptively to determine group means and standard deviations for the measures. The normality of data was verified using a Shapiro–Wilk test. The results of the normality test demonstrate that the data came from a normal distribution of motor control and attention control parameters (ranging from 0.056 to 0.670). In addition, the mixed model ANOVA was implemented to compare the measured data at pre-test and post-test between the groups and measured within a factor of time and between groups and interaction effect. Additionally, to examine if the effect of drum playing with rhythmic cueing varied depending on the intervention between groups of parameters, the effect size estimate was analyzed using a Cohen’s d measure. An effect size of 0.2–0.4 was considered a low effect size, with 0.5–0.7 was a moderate effect size, and more than 0.8 was considered a large effect size.

## 3. Results

In this study, the mean age of the participants was 62.16 years and the mean duration of disease was 5.25 years. In addition, there were no significant differences between the two groups in terms of age, duration of disease, education level, the H&Y scale, and the K-MMSE (see Table 3).

Changes in the mean and standard deviation of the data from pre-test to post-test for each group and effect size of parameters between groups are displayed in Table 4. The motor control measures, including the MIDI-drum tapping task and NHPT, and the cognitive measures are presented.

**Figure 3:**
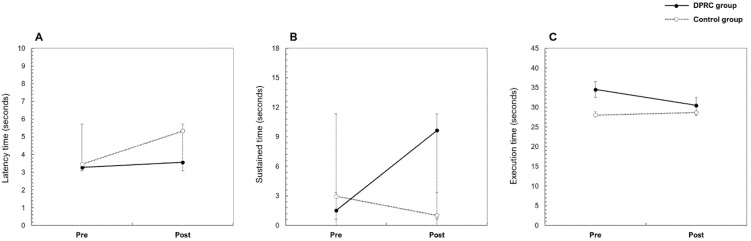
Changes in motor control measures for the DPRC and control groups. Panel (**A**) shows change in latency time until entrainment for 105 BPM, panel (**B**) shows change in sustained time of entrainment for 45 BPM, and panel (**C**) shows change in execution time of left hand for NHPT.

In addition, a mixed model ANOVA was conducted to compare the DPRC group and control group in terms of changes in each measure across time. The results are displayed in Table 5. There were significant time effects in motor control measures in the condition of the latency time until entrainment at 105 BPM, sustained time of entrainment at 45 BPM, and execution time of the left hand for which large effect sizes were measured in Table 4. These results indicate that changes in measures were significant across time. According to the results for latency time until entrainment at 105 BPM, both groups showed increases at post-test. And for the sustained time of entrainment at 45 BPM, only the DPRC group showed increases at post-test. Additionally, there were significant time effects in execution time on the NHPT for the left hand. Both groups showed decreases in execution time at post-test (see Figure 3).

Additionally, significant group effects in changes at posttest compared to the pretest were observed in motor control measures in the condition of the latency time until the entrainment in 105 BPM for which large effect size was measured in Table 4. While the DPRC group exhibited an increase in latency time following entrainment at 105 BPM, the control group showed a greater increase in this latency time. In particular, a significant interaction effect for time and group was observed for latency time until entrainment at 105 BPM (see Table 5).

## 4. Discussion

We investigated the effects of DPRC on the motor control and cognitive functioning of patients with PD. Results showed that the latency time before self-paced tapping and the latency time before tapping entrainment in response to rhythmic cueing did not differ between groups. Meanwhile, changes in tempo matched to movement resulted in changes in dual-task performance. Significant differences were found between the groups for the condition of sustained movement at a slow tempo. Along with these results, we found that bimanual DPRC yielded changes in attention control as manifested by improvement in inhibitory control during cognitive processing.

It is remarkable that DPRC improved performance on the cognitively demanding dual tasks. Each step of drum playing involves regular cueing with temporal alternations, which helps patients with PD practice focusing more attention on bimanual motor execution following auditory external cues. As several previous studies suggest, temporal preparation effects driven by rhythmic cueing could be presented during drum playing with cueing [51,52]. Besides the rhythmic aspect, various musical elements, such as pitch and timbre, in intervention contexts provide complex perceptual information as auditory input. With this auditory-motor interaction during drum playing, higher level cognitive processing may be stimulated. This is shown in the MIDI drum tapping task results indicating that significant differences exist between sustained time for movement entrainment in rhythmic cueing at slow tempo. Moving at slower tempo requires tempo adjustment to external auditory cues with a higher demand on cognitive functions [53,54,55]. This implies that bimanual DPRC facilitates intense auditory motor interaction requiring high-level cognitive processing during bimanual tasks for patients with PD.

In particular, we found a significant effect for slow tempo (BPM = 45) on sustained time for movement entrainment in the DPRC group. This task of sustaining regular intervals and continuing bimanual motor performance requires more control in the timing and speed of bimanual performance compared to simpler motor tasks without auditory cues, such as sequential finger tapping movements [56]. Thus, slow and repetitive movement tasks can be considered key to dual-task training for PD patients by enhancing time-keeping abilities during auditory-motor interaction [32,57,58,59,60]. Additionally, this supports that temporal characteristics in rhythmic cueing serve as movement timing cues to activate the motor system with attention control [61]. We also found significant improvement in left hand scores of the NHPT. Compared to the average scores of hand function tests reported in previous studies of PD patients [62], this result suggests that DPRC was effective in producing improvements in non-dominant hand function. Overall, the results indicate that regular intervals with external auditory cues constitute an internal timekeeping system and that this mechanism can improve perceptual accuracy of timing and thus be effectively applied to interventions targeting sensorimotor functioning and motor coordination.

Given the impairment in frontal cortical activity due to basal ganglia dysfunction in PD [63], executive function during repetitive movement plays an important role in controlling movement performance [64]. Our data point to independence between hand function or simple motor performance abilities and functional changes in cognitive processing. Considering the movement of bimanual drum playing, the level of movement involved in the task is simultaneous and simple up-down upper extremity movement without individual finger movements associated with dexterity. Further studies should be conducted with various musical instruments or more complex rhythmic patterns in order to substantiate both cognitive and motor improvements.

This study is not without limitations. First our sample size was small and our results should be considered with caution. Although randomization was employed in the study design, we could not blind the intervention due to the nature of music intervention studies. Future studies should be designed as long-term studies and involve rigorous data collection on executive function considering the specific neurological impairments of patients with PD.

## 5. Conclusions

In sum, the current study provides interesting findings revealing the effects of bimanual DPRC on enhancing attention controls for patients with PD. Regular rhythmic cueing has the power to enhance temporal preparation during temporal changes are made during motor tasks, particularly when temporal direction is getting slower. Overall, results unveil instrument playing with rhythmic cueing based on a dual-task paradigm can facilitate simultaneous motor and attention control functions and meaningful changes in dual-task performance outcomes for patients with PD. Variation of tempi in rhythmic cueing and complexity of intervention tasks affect motor control function, attention control, and demand on cognitive processing. Bimanual drum playing as a musical application can be used as an efficient rehabilitative intervention modality for patients with PD. The dual-task paradigm should continue to be applied in rehabilitative intervention contexts.

## Figures and Tables

**Table 1:** The MIDI drum tapping task.

Tapping Task		Cueing	Condition of Cueing
Without rhythmic cueing	Tapping to own pace	No	-
With rhythmic cueing	Tapping to follow regular rhythmic cueing	Yes	Providing regular rhythmic cueing for 15 s
	Shifting tapping to match slow and fast tempo	Yes	Changing tempo of regular rhythmic cueing after 15 s
	Sustained tapping to regular intervals with cueing removed	Y→N	Removing regular rhythmic cueing after 15 s

**Table 2:** Intervention of drum playing with rhythmic cueing.

Stage Intervention Task		Regular Cueing	Interrupted Cueing	Content
1	Drum playing with regular rhythmic cueing	Y	N	Tracking rhythmic cueing of regular interval of metronome beats
2	Drum playing with alternating tempo	Y	N	Tracking rhythmic cueing according to changes in tempo of 45 and 105 BPM
3	Drum playing with regular rhythm and unexpected stimulus interruption	Y	Y	Sustained regular interval of rhythmic cueing with unpredicted melodic cueing
4	Task shifting between initiation and stop with rhythmic cueing of different rhythmic patterns	Y	Y	During tracking of rhythmic cueing for regular tempo, participants shift tasks from initiating to stopping when provided with cueing of different rhythmic patterns
5	Task shifting between playing and singing with different rhythmic patterns	Y	Y	During tracking of rhythmic cueing for regular tempo, participants shift tasks between playing and singing when provided with different rhythmic patterns

**Table 3:** Participants’ demographic information.

Characteristic	DPRC *M ± SD*	Control *M ± SD*	*Z*	*p*
Age (years)	61.6 ± 4.9	63.1 ± 10.1	−0.434	0.673
Duration of disease (years)	5.6 ± 3.1	4.8 ± 1.4	1.000	0.363
Education (years)	12.3 ± 1.2	12.3 ± 3.1	−0.416	0.686
H&Y scale	2.3 ± 0.8	1.6 ± 0.5	0.455	0.660
MMSE-K score	28.3 ± 1.2	26.3 ± 1.9	1.570	0.132

Note. DPRC: drum playing with rhythmic cueing group; Control: control group; H&Y Scale: the Hoehn and Yahr scale; MMSE-K: the Korean version of the Mini-Mental State Examination.

**Table 4:** Changes in motor control and cognitive measures for each group.

Parameter		DPRC Group (*n* = 6)		Control Group (*n* = 6)		
		Pre *M ± SD*	Post *M ± SD*	Pre *M ± SD*	Post *M ± SD*	Effects Size
**Motor control measures**						
Latency time until regularly self-paced tapping	Self-paced	0.84 ± 0.37	0.55 ± 0.23	0.71 ± 0.52	0.96 ± 0.2	0.94
Latency time until entrainment	45 BPM	4.05 ± 3.04	4.41 ± 2.78	3.87 ± 2.9	8.49 ± 4.31	0.53
	105 BPM	3.27 ± 0.65	3.56 ± 1.25	3.46 ± 3.14	5.33 ± 3.88	1.46
Latency time until entrainment for changes in tempo	45→105 BPM	4.01 ± 4.59	1.93 ± 0.4	4.75 ± 4.31	5.64 ± 4.32	0.84
	105→45 BPM	4.91 ± 2.78	3.59 ± 2.87	4.49 ± 1.03	7.03 ± 3.75	0.39
Sustained time of entrainment	45 BPM	1.52 ± 2.19	9.65 ± 5.56	2.96 ± 3.92	1.03 ± 1.73	0.9
	105 BPM	2.55 ± 1.3	7.12 ± 5.23	2.17 ± 1.18	3.11 ± 2.45	0.68
Execution time of NHPT	Right	28.78 ± 6.14	27.64 ± 6.39	29.42 ± 5.45	39.60 ± 8.68	0.03
	Left	34.51 ± 11.99	30.47 ± 8.84	28.00 ± 4.58	28.69 ± 1.71	0.87
**Cognitive measures**						
K-TMT-e_A		19.16 ± 5.41	19.18 ± 4.92	21.12 ± 5.07	21.94 ± 5.65	0.16
K-TMT-e_B		42.67 ± 18.06	33.68 ± 13.40	41.06 ± 15.79	33.43 ± 8.56	0.07
KST_WR_Time		65.55 ± 8.46	65.88 ± 15.71	63.51 ± 10.46	65.27 ± 10.58	0.14
KST_CR_Time		121.69 ± 34.13	109.73 ± 26.86	119.08 ± 21.38	129.70 ± 27.25	1.04

Note. DPRC: drum playing with rhythmic cueing group; Control: control group; BPM: beats per minute; K-TMT-e: Korean trail making test for the elderly; KST: Korean Stroop test; WR: word reading condition; CR: color reading condition.

**Table 5:** The results of a mixed model repeated measures ANOVA.

Parameter		Repeated Measures Results		
		Time Effect	Group Effect	Time * Group
		*(t, p)*	*(t, p)*	*(t, p)*
**Motor control measures**				
Latency time until regularly self-paced tapping	Self-paced	0.011, 0.917	0.764, 0.403	2.704, 0.131
Latency time until the entrainment	45 BPM	2.686, 0.132	0.178, 0.682	0.873, 0.372
	105 BPM	6.231, 0.032 **	5.407, 0.042 **	6.469, 0.029 **
Latency time until the entrainment in changes tempo	45→105 BPM	1.214, 0.296	0.939, 0.355	0.477, 0.505
	105→45 BPM	1.094, 0.320	0.990, 0.343	2.124, 0.176
Sustained time of entrainment	45 BPM	8.926, 0.014 **	0.017, 0.900	2.448, 0.149
	105 BPM	3.130, 0.107	0.031, 0.864	1.414, 0.262
Execution time of NHPT	Right	0.284, 0.606	0.035, 0.856	0.004, 0.954
	Left	10.994, 0.008 ***	0.110, 0.747	2.327, 0.158
**Cognitive measures**				
K-TMT-e_A		0.083, 0.779	0.616, 0.451	0.078, 0.786
K-TMT-e_B		2.302, 0.160	0.016, 0.901	0.015, 0.903
KST_WR_Time		0.132, 0.724	0.038, 0.849	0.062, 0.808
KST_CR_Time		0.011, 0.917	0.279, 0.609	3.295, 0.100

Note. * shows the interaction effect. ** *p* < 0.05. *** *p* < 0.01. DPRC, drum playing with rhythmic cueing group; control, control group; sec, seconds, BPM, beats per minute. K-TMT-e, Korean trail making test for the elderly (Yi et al., 2007); KST, Korean Stroop test (Kim et al., 2004); WR, word reading condition; CR, color reading condition.
